# Hemodynamic Profiles of Cardiogenic Shock Depending on Their Etiology

**DOI:** 10.3390/jcm9113384

**Published:** 2020-10-22

**Authors:** Mélanie Gaubert, Marc Laine, Noémie Resseguier, Nadia Aissaoui, Etienne Puymirat, Gilles Lemesle, Pierre Michelet, Sami Hraiech, Bruno Lévy, Clément Delmas, Laurent Bonello

**Affiliations:** 1 Cardiology Department, APHM, Mediterranean Association for Research and Studies in Cardiology (MARS Cardio), Centre for CardioVascular and Nutrition Research (C2VN), Aix-Marseille Univ, INSERM 1263, INRA 1260, Hopital Nord, 13015 Marseille, France; melanie.gaubert@ap-hm.fr (M.G.); marc.laine@ap-hm.fr (M.L.); 2 Support Unit for Clinical Research and Economic Evaluation, APHM, 13385 Marseille, France; noemie.RESSEGUIER@univ-amu.fr; 3 Department of Critical Care Unit, Assistance Publique-Hôpitaux de Paris (AP-HP), Hôpital Européen Georges Pompidou (HEGP), Université Paris-Descartes, 15015 Paris, France; nadia.aissaoui@egp.aphp.fr; 4 Département de Cardiologie, Hôpital Européen Georges Pompidou, Assistance Publique des Hôpitaux de Paris, 75015 Paris, France; etienne.puymirat@aphp.fr; 5 USIC et Centre Hémodynamique, Institut Coeur Poumon, Centre Hospitalier Régional et Universitaire de Lille, Faculté de Médecine de l’Université de Lille, Institut Pasteur de Lille, Unité INSERM UMR 1011, and FACT (French Alliance for Cardiovascular Trials), F-59000 Lille, France; gilles.lemesle@chru-lille.fr; 6 Service d’accueil des Urgences, Hopital Timone, 13005 Marseille, France; pierre.michelet@mail.ap-hm.fr; 7 Resuscitation Department, Aix-Marseille Univ, APHM, Hôpital Nord, 13005 Marseille, France; sami.hraiech@ap-hm.fr; 8 CHRU Nancy, Service de Réanimation Médicale Brabois, Pôle Cardiovasculaire et Réanimation Médicale, Hôpital Brabois, 54511 Vandoeuvre les Nancy, France; b.levy.chru-nancy@apicrypt.fr; 9 INSERM UMR-1048, Intensive Cardiac Care Unit, Rangueil University Hospital, 31400 Toulouse, France; delmas.clement@chu-toulouse.fr

**Keywords:** cardiogenic shock, hemodynamic evaluation, acute myocardial infarction, acute decompensated heart failure

## Abstract

The pathophysiology of cardiogenic shock (CS) varies depending on its etiology, which may lead to different hemodynamic profiles (HP) and may help tailor therapy. We aimed to assess the HP of CS patients according to their etiologies of acute myocardial infarction (AMI) and acute decompensated chronic heart failure (ADCHF). We included patients admitted for CS secondary to ADCHF and AMI. HP were measured before the administration of any inotrope or vasopressor. Systemic Vascular Resistances index (SVRi), Cardiac Index (CI), and Cardiac Power Index (CPI) were measured by trans-thoracic Doppler echocardiography on admission. Among 37 CS patients, 28 had CS secondary to ADCHF or AMI and were prospectively included. The two groups were similar in terms of demographic data and shock severity criteria. AMI CS was associated with lower SVRi compared to CS related to ADCHF: 2010 (interquartile range (IQR): 1895–2277) vs. 2622 (2264–2993) dynes-s·cm^−5^·m^−2^ (*p* = 0.002). A trend toward a higher CI was observed: respectively 2.13 (1.88–2.18) vs. 1.78 (1.65–1.96) L·min^−1^·m^−2^ (*p* = 0.067) in AMICS compared to ADCHF. CS patients had different HP according to their etiologies. AMICS had lower SVR and tended to have a higher CI compared to ADHF CS. These differences should be taken into account for patient selection in future research.

## 1. Introduction

Cardiogenic shock (CS) remains a major clinical challenge with a stable incidence and a high mortality [1,2,3]. Because of its complex pathophysiology and the limited evidence-based therapeutic interventions, the current therapeutic strategy are supported by a low level of evidences. CS involves a profound depression of myocardial contractility considered to trigger hemodynamic instability, resulting in a potentially deleterious spiral of reduced cardiac output, low blood pressure, and further coronary ischemia. In addition, a dysfunction of the entire circulatory system, through inadequate circulatory compensation, also plays a critical role in the occurrence of CS [4,5]. Evidences suggest that there is a large variability in systemic vascular resistances (SVR) and a wide range of hemodynamic profiles in CS [6].

Although acute myocardial infarction (AMI) remains a major etiology of CS, acute decompensated chronic heart failure (ADCHF) is a growing cause and may be the leading cause nowadays [2,7,8,9]. Together, these two etiologies represent the large majority of CS etiologies [9]. Because ADCHF patients experiencing CS present with hemodynamic conditions and neurohormonal milieu that are often strikingly different from patients with CS related to AMI, they may have different hemodynamic profiles and thus require specific treatments [5].

Accurate hemodynamic characterization may therefore be critical to improve the therapeutic strategy including catecholamine choice in CS. Systemic vascular resistance index (SVRi) and Cardiac Power Index (CPI) constitute critical diagnosis and prognosis parameters in CS and can easily be assessed non-invasively by transthoracic echocardiography (TTE) [6,10,11].

We sought to compare the hemodynamic profiles of patients presenting with CS related to AMI and those with CS related to ADCHF.

## 2. Experimental Section

### 2.1. Patients

This prospective single-center study was conducted in the ICU of our institution (University Hospital, Hopital Nord, Marseille, France), after approval by the ethical committee of our institution, between December 2017 and September 2018. The CPP (French ethical committee) number for this assay is 17-09-95. All CS patients admitted in our ICU were screened for enrolment. Only CS secondary to ADCHF and to AMI were included after an informed consent was obtained. All patients gave written informed consent before inclusion (ClinicalTrials.gov: NCT03283995). The main non-inclusion criteria included (1) CS caused by arrhythmias, toxic, or post-partum; (2) an inadequate acoustic window for echocardiography; (3) Pre-hospital treatment with inotrope or vasopressor.

### 2.2. Definitions

CS was defined according to guidelines as systolic blood pressure <90 mm Hg despite adequate intravascular volume, and clinical or laboratory signs of hypoperfusion (cold extremities, oliguria, mental confusion, dizziness, narrow pulse pressure, metabolic acidosis, elevated serum lactate, or acute kidney failure) [12]. AMI was defined as a clinical setting consistent with acute myocardial ischemia (symptoms of ischemia, new or presumed new significant ST-T wave changes or left bundle branch block on 12-lead ECG) associated with a significant increase and/or decrease of high-sensitivity cardiac troponin, with at least one value above the 99th percentile of the upper reference limit. In addition, the finding of a culprit lesion during coronary angiography was required [13]. ADCHF was defined as typical symptoms or sign (breathlessness, elevated jugular venous pressure, pulmonary crackles, and peripheral edema) caused by a pre-existing structural and/or functional cardiac abnormality, resulting in a reduced cardiac output and/or elevated intracardiac pressures [12].

### 2.3. Measurements

Hemodynamic evaluation was performed by trained cardiologists on admission before any catecholamine infusion. Systemic vascular resistance index (SVRi) was determined using mean arterial pressure (MAP), right arterial pressure (RAP), and cardiac index (CI) as: SVRi (dynes.sec·m^2^·cm^−5^) = (MAP − RAP) (mmHg) × 80/CI (L·min^−1^·m^−2^) [10]. CI was calculated using the velocity–time integral (VTI) of the left ventricular outflow tract (LVOT) in the apical views, the diameter of the LVOT measured in parasternal long axis, and the heart rate (HR). Thus, CI = Cross sectional area (cm^2^) × VTI (cm) × HR/body surface area. RAP was estimated on the basis of inferior vena cava size and its breathing-related collapsibility according to guidelines: size ≤2.1 cm and collapses >50% during sniff test = RAP 0–5 mm Hg; Size > 2.1 cm and collapses >50% during sniff = RAP 5–10 mmHg; Size >2.1 cm and collapses <50% during sniff = RAP 10–20 mm Hg [9]. CPI was calculated as CPI × MAP [6].

### 2.4. Statistical Analysis

Clinical characteristics and hemodynamic measurements were described using numbers and percentages for qualitative variables and using medians and interquartiles for quantitative variables. Comparisons between groups for qualitative variables were performed using the χ² test, or the nonparametric Fisher’s test as appropriate, and for quantitative variables using the Kruskal–Wallis test and the Mann–Whitney test. All statistical analyses were performed using R software version 3.4.1 (company foundation for statistical computing, Vienna, Austria). All tests were two-sided, and a *p*-value < 0.05 was considered statistically significant.

## 3. Results

### 3.1. Population

**Figure 1:**
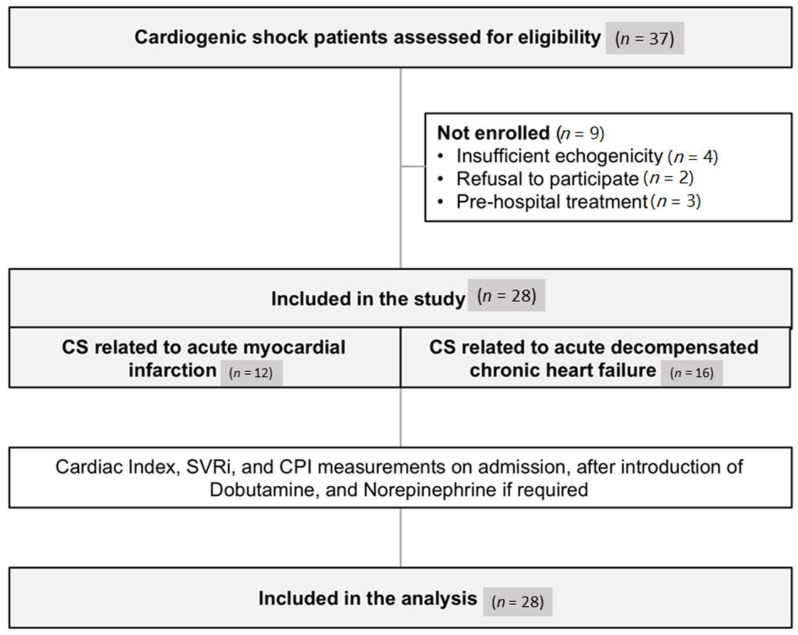
Flow chart of the study. CS, cardiogenic shock; SVRi, Systemic vascular resistance index; CPI, cardiac power index.

Thirty-seven consecutives patients admitted for CS related to AMI or ADCHF during the inclusion period were assessed for eligibility. Among them, 28 patients were finally included. Non-inclusion was related to an insufficient echogenicity (4 patients), prehospital inotropic support (3 patients), and refusal to participate (2 patients) (Figure 1).

Baseline characteristics of the 28 patients included are presented in Table 1. CS was related to AMI in 12 patients and to ADCHF in 16 patients. All patients received dobutamine as first line and 10 (35.7%) required the addition of norepinephrine because they failed to reach the therapeutic goal of a mean arterial pressure of 65 mmHg.

### 3.2. Hemodynamic Profiles

**Figure 2:**
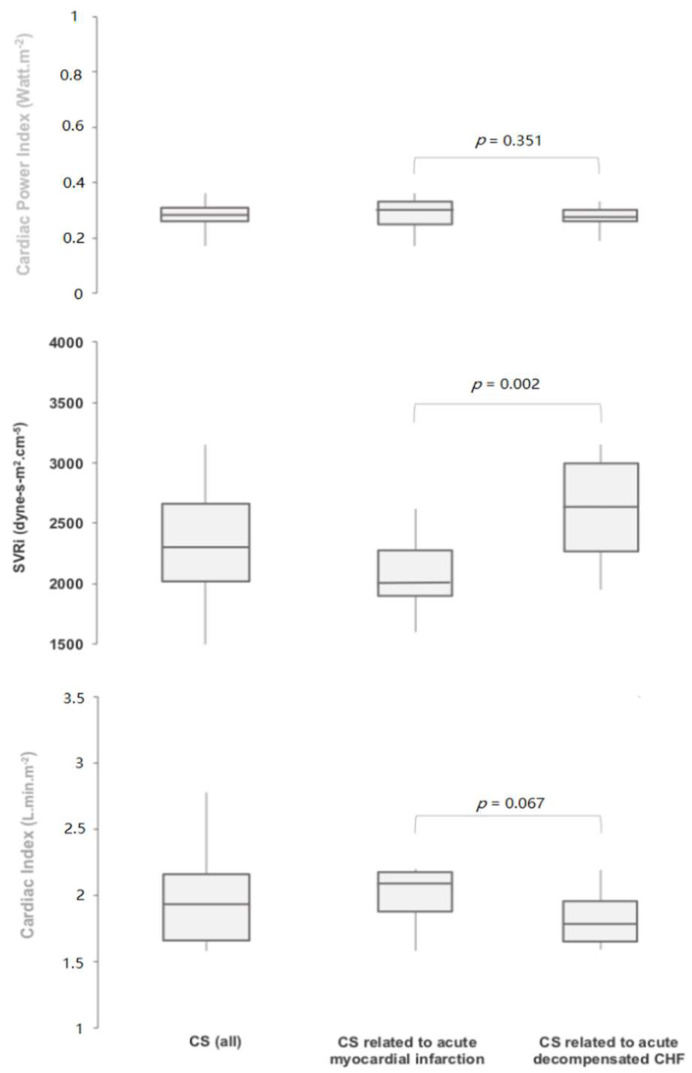
Hemodynamic profile on admission. CHF, Chronic heart failure; CS, Cardiogenic shock; SVRi, Systemic vascular resistance index.

Hemodynamic profiles on admission are displayed in Figure 2 and Table 1. In CS patients, MAP on admission was 67.1 ± 8.8 mmHg, mean left ventricular ejection fraction was 26 ± 9%, and mean cardiac index was 1.92 ± 0.28 L·min^−1^·m^−2^. CS related to AMI was associated with significantly lower median SVRi than CS related to decompensated heart failure: 2010 (IQR 1895–2277) vs. 2622 (2264–2993) dynes-s·cm^−5^·m^−2^, respectively (*p* = 0.002), and a trend toward a higher CI: respectively 2.13 (1.88–2.18) vs. 1.78 (1.65–1.96) L·min^−1^·m^−2^ (*p* = 0.067). However, CPI remained identical in the 2 groups: respectively 0.3 (0.26–0.31) vs. 0.27 (0.26–0.30) Watt·m^−2^ (*p* = 0.351).

### 3.3. Outcome

All patients received dobutamine as first-line agent. Norepinehrine was required in about one-third of patients in both groups. In-hospital mortality was identical in the 2 groups (25%). The overall survival at 1-month follow-up was 64.3%.

## 4. Discussion

In the present study, the HP of CS related to AMI and ADCHF differed significantly. CS related to AMI was associated with significantly lower SVRi and a trend toward a higher CI compared to those related to an ADCHF. These findings are original and of potential clinical interest to better tailor therapy for future studies assessing therapeutic strategies in CS. Indeed, these differences underline the need to accurately select patients according to the etiology of CS in future trials evaluating new therapeutic strategies. In addition, our findings also suggest that the first line catecholamine may differ depending on CS etiology.

In line with our findings, Cotter et al. reported a large heterogeneity of hemodynamic profiles in CS patients, with relatively elevated SVR in some patients and very low SVR in others. However, in his study, the number of patients with CS was limited and the relation with the etiology of CS was not described [6]. Our results further suggest that, despite similar CPI, CS patients have different HP in relation with their etiology. Although a low CI is a part of CS definition, the cut-off of the CI may be variable (Refer to CS review Holger Thiele EHJ) and various hemodynamic measures were reported [5]. Consistent with our findings, low SVRi in CS related to AMI were observed in the SHOCK trial [14]. Such low SVR were related to systemic inflammation in relation with myocardial necrosis triggered by interleukins, tumor necrosis factor-alpha, and inducible nitric oxide synthase [4,15,16]. Although we did not assess these biomarkers, previous studies have observed a rise in plasma levels of inflammatory cytokines after AMI, which were associated with mortality [17,18]. On the other hand, in patients with chronic heart failure, the profound upregulation of vasoconstrictor substances such as angiotensin II, endothelin-1, and norepinephrine may explain the relatively preserved compensatory vasoconstriction observed in this group in our study [19].

Our results are original and of potential clinical interest in the setting of CS were improvements are slow and studies scarce. It is widely acknowledged that the selection of patients for a therapeutic intervention is very challenging in CS, but appears critical to adequately evaluate its benefit. The results of the present study suggest that future clinical trials should take into account the etiology of CS, because it is associated with a specific HP. This is particularly relevant in studies focusing on catecholamines in CS. This would allow a more uniform selection of patients and an accurate evaluation of the dedicated therapeutic intervention.

There are some limitations to the present study. Hemodynamic evaluations were performed using doppler echocardiography and invasive measures were not performed according to the current practices in our ICU. Of note, we recently showed a strong correlation between doppler echocardiography and transpulmonary thermodilution in hemodynamic evaluation in patients presenting with CS. [20] Furthermore, current guidelines already support the use of transthoracic echocardiography to provide hemodynamic monitoring in these settings [12,21]. In addition, the relatively small sample size does not allow to differentiate subgroups of patients such as those with left main trunk occlusion who may present a different hemodynamic profile. Although the main limitations of the present study are related to its monocentric design and the low number of patients, the prospective and consecutive inclusion limited the likelihood of bias.

## 5. Conclusions

In the present study, we identified different HP of CS depending on their etiology. CS related to AMI has significantly lower SVR compared to CS secondary to ADCHF. This specific hemodynamic profile should be taken into account for patient selection in future research and therapeutic strategy optimization including catecholamine choice.

## Figures and Tables

**Table 1:** Clinical, biological, and angiographic characteristics of the study population and hemodynamic profile on admission.

	CS Related to AMI (*n* = 12)	CS Related to ADCHF (*n* = 16)	*p* Value
Age, Years	72 (61–81)	78 (66–80)	-
Male Gender	9 (75%)	13 (81%)	-
BMI	26.3 (24.4–27.7)	25.5 (20.7–28.2)	-
SOFA Score	6 (5–7)	6 (4–8)	-
**Cardiomyopathy**			-
Ischemic	12 (100%)	6 (38%)	-
Valvular	0	5 (31%)	-
Dilated	0	3 (19%)	-
Pulmonary heart disease	0	1 (6%)	-
Toxic	0	1 (6%)	-
**Medical History**			
PCI	8 (67%)	8 (50%)	-
CAB	2 (17%)	0	-
Hypertension	9 (75%)	9 (56%)	-
Diabetes	7 (58%)	5 (31%)	-
Dyslipidaemia	3 (25%)	1 (6%)	-
Smoking	7 (58%)	6 (38%)	-
**Biology**			
Leucocytes (G/L)	12.0 (7.4–13.9)	7.8 (7.0–9.3)	-
Troponin (µg/L)	9.5 (0.2–76.0)	0.1 (0.1–0.2)	-
Bilirubin (µmol/L)	20.0 (7.8–28.3)	27.5 (17.8–38.5)	-
Cr Cl (mL/min)	48.1 (36.7–67.7)	47.0 (35.1–61.5)	-
Lactates (mmol/L)	2.0 (1.7–2.4)	2.1 (1.6–2.5)	-
CRP (mg/L)	44.5 (13.6–107.3)	25.9 (18.5–38.8)	-
**Coronary Status**			
**Coronary Angiography**	12 (100%)	−	-
**Number of Treated Vessel**			
1	2 (17%)	−	-
2	5 (42%)	−	-
3	5 (42%)	−	-
**Treatment**			
Dobutamine	12 (100%)	16 (100%)	-
Norepinephrine	4 (33%)	6 (38%)	-
Diuretic	9 (75%)	16 (100%)	-
**Evolution**			
ICU length of stay, days	7.5 (4.8–10.0)	9.0 (5.8–10.0)	-
Hospital survival	9 (75%)	12 (75%)	-
30 days survival	9 (75%)	9 (56%)	-
**Hemodynamic Profile on Admission**			
MAP (mmHg)	62.5 (58.8–70.5)	70.0 (66.0–75.0)	0.1
LVEF (%)	30 (25–30)	23 (15–30)	0.4
Cardiac Index (L·min^−1^·m^−2^)	2.13 (1.88–2.18)	1.78 (1.65–1.96)	0.06
SVRi (dynes-s-m^2^·cm^−5^)	2010 (1895–2277)	2622 (2264–2993)	<0.01
CPI (Watt·m^−2^)	0.30 (0.25–0.33)	0.27 (0.26–0.30)	0.4
LVEF (%)	30 (25–30)	23 (15–30)	0.4
RAP (mmHg)	11 (8.75–15)	13 (10–15)	0.91

Results are expressed as median (interquartile range) or as number (%).CS, Cardiogenic Shock; BMI, Body Mass Index; SOFA, Sepsis-related Organ Failure Assessment; PCI, Percutaneous Coronary Intervention; CAB, Coronary Artery Bypass; Cr Cl, Creatinine Clearance; CRP, C Reactive Protein; ICU, Intensive Care Unit. LVEF, Left Ventricular Ejection Fraction; MAP, Mean Arterial Pressure; SVRi, Systemic vascular Resistance index; CPI, Cardiac Power Index; RAP, Right Arterial Pressure.
